# Accurate Determination of Boron Content in Halite by ICP-OES and ICP-MS

**DOI:** 10.1155/2019/9795171

**Published:** 2019-05-29

**Authors:** Zhang-kuang Peng, Zhi-na Liu

**Affiliations:** ^1^School of Earth Sciences and Resources, China University of Geosciences, 100083 Beijing, China; ^2^No. 208 Geologic Party, CNNC, Baotou 014010, Inner Mongolia, China

## Abstract

Boron element is widely distributed in different geologic bodies, and there are important geo-chemical applications in earth science. Halite is a common mineral found in sediment basin. However there is no good method to accurately measure the boron content in halite, which is mainly because Inductively Coupled Plasma Optical Emission Spectrometer (ICP-OES) and Inductively Coupled Plasma Mass Spectrometer (ICP-MS) are limited by the high salt matrix interference and the instrument detection limit. Thus enriching the boron element and removing the matrix interference are necessary before the measuring. In this paper, Amberlite IRA 743 boron-specific resin was applied to enrich the boron element and remove most of the high-salt matrix. The strong acid cation resin (Dowex 50 W×8, 200-400 mesh, USA) and weak-base anion resin (Ion Exchanger II, Germany) were mixed with equal volume, which could remove the foreign ions completely: meanwhile, the relative content of boron in the solution reached above 98%, and the recoveries ranged from 97.8% to 104%. 208.900 nm was chosen as the detection wavelength for ICP-OES, and the detection identification and quantification limits were 0.006 mg·L^−1^ and 0.02 mg·L^−1^, respectively. ^11^B was chosen as the measuring element for ICP-MS, and the detection identification and quantification limits were severally 0.036 mg·L^−1^ and 0.12 mg·L^−1^. The relative standard deviations ranged from 1.4% to 3.4% through six replicates under different salinities. Therefore, the process could be regarded as a feasible method to measure boron content in halite by ICP-OES and ICP-MS.

## 1. Introduction

The boron is a strongly incompatible element in the earth [1], which makes the boron remain in the solution during the evaporation, and, with the development of evaporation, the boron content in the halite increases [2, 3]. As the boron gets into the halite in the form of inclusion, it could reflect the salinity of the paleo-lake water as well as drier paleoclimatic conditions. The geo-chemical behavior of boron in halite is a useful tool to study the evolution and chemical composition of salt lake [2–5]. However, there were few reports about measuring the boron content in halite. Spectrophotometry is a common method for the determination of boron content in many minerals, such as biological sample, soil, plants, and food [6–11]; meanwhile it was the only method to measure the boron content in boron-rich halite formed in laboratory [4]. Due to the low sensitivity and serious matrix interference, it is not good for measuring the boron content in natural halite.

Because of the high sensitivity and rapid analysis, the ICP-MS and ICP-OES are good methods for measuring the boron content in different minerals, such as coal, quartz, and other geochemical samples [12–15]. However, there was no report about using ICP-MS and ICP-OES to measure the boron content in halite. Though dilution is the commonest method to overcome the high-salt matrix interference, it is not for measuring the boron content in halite because it will cause the boron content in the solution to fall below the detection limit of the instrument. So preenrichment of boron element and reducing the salinity from solution are the keys to successfully measure the boron content in halite by ICP-OES or ICP-MS. In this paper, Amberlite IRA 743 boron-specific resin was used to enrich boron and remove most of the NaCl. Then elute the adsorbed boron from the resin with hydrochloric acid, whose volume, content, and temperature were 10 mL, 0.1 mol·L^−1^, and 75°C, respectively. The conditions of eluent were reported by Xiao et al., who found that the boron was hard to be eluted from the boron-special resin even if the concentration of HCl reached 2 mol·L^−1^, while it can be easily eluted by HCl in high temperature [16]. The strong acid cation resin (Dowex 50 W×8, 200-400 mesh, USA) and weak-base anion resin (Ion Exchanger II, Germany) were mixed with equal volume to remove NaCl and HCl. Finally send the solution to measure boron content by ICP-OES and ICP-MS.

## 2. Material and Methods

### 2.1. Apparatus

Inductively Coupled Plasma Optical Emission Spectrometry (ICP-OES, ICAP 6500 DUO, Thermo Electron): RF power was 1150 W; flux of cooling air was 15 L·min^−1^; flux of auxiliary air was 0.5 L·min^−1^; flux of carrying-air was 0.55 L·min^−1^; pump speed was 60 r·min^−1^; observation way of plasma was automatic.

Inductively Coupled Plasma Mass Spectrum (ICP-MS, X series 2 type, Thermo Electron): RF power was 1250 W; flux of cooling air (Ar) was 12.0 L·min^−1^; flux of auxiliary was 0.75 L·min^−1^; atomizer flow (Ar) was 0.85 L·min^−1^; measuring method was peak jumping with ^11^B.

### 2.2. Reagents

Boric acid solid (H_3_BO_3_, GR) and sodium chloride solid (NaCl, GR) were made in Beijing Reagent Factory. Balanced hydrochloric acid was made from concentrated hydrochloric acid (GR): sub-boiling ammonia. Deionized water was made through multiple distillation and then the boron was removed by Amberlite IRA 743 type boron-specific resin made in American Rohm & Hass Company. It contains hydrophobic styrene skeleton and tertiary amine group and can strongly adsorb borate anion from alkaline solution with an exchange capacity of 10.9 mg B•g^−1^ [17]. Mixed resin was made of an equal volume of strong acid cation resin (Dowex 50 W×8, 200-400 mesh, USA) and weak-base anion resin (Ion Exchanger II, Germany).

Natural Samples: natural salt samples came from ZK309 Drill Hole, Long-hu Diggings, Laos (ZK-03, ZK-04, ZK-10), and salt lake in Pakistan (KR03-2, BS01-2, KS05).

### 2.3. Certified Reference Materials, Samples, and Sample Preparation

Prepare a standard solution of 10 mg·L^−1^ boron, and then use it and NaCl to make a series of mixtures with boron content from 10 *μ*g to 70 *μ*g and NaCl from 500 mg·L^−1^ to 50000 mg·L^−1^. Because the boron was only adsorbed by boron-special resin in the form of B(OH)_4_^−^ in the alkaline solution, the sub-boiling ammonia is used to adjust the pH of the solution to 7~8 [18], and then the purification process of boron-specific resin is carried out by previous studies [7]. Boron is eluted from the resin with 10 mL 0.1 mol·L^−1^ HCl at 75°C, and then mix the strong acid cation resin (Dowex 50 W×8, 200-400 mesh, USA) and weak-base anion resin (Ion Exchanger II, Germany) with equal volume to remove the foreign ions from the eluent. Finally determine boron content by ICP-OES and ICP-MS. During step one, the boron is adsorbed by boron-special resin in the form of B(OH)_4_^−^. Next the foreign ions are adsorbed by the mixed resins in which the boron exists in the form of H_3_BO_3_ in solution, and it is separated from foreign ions.

For numbers ZK-03, ZK-04, ZK-10, KR03-2, BS01-2, and KS05, weight 5.0 g halite and dissolve them into 50 mL deionized water. Remove the high-salt matrix and enrich the boron element using the above method.

## 3. Result and Discussion

### 3.1. The Result of Boron Recovery

**Figure 1:**
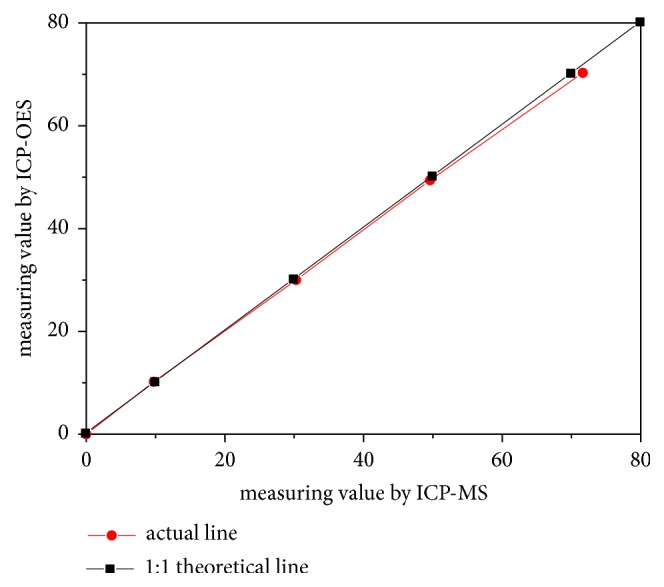
The relationship of measuring value by ICP-OES and ICP-MS. The lines of actual values were consistent with the theoretical line of 1:1 which showed that the values measured by ICP-OES were the same as those by ICP-MS.

The recovery of boron in the pure solutions ranged from 97.6% to 102.34% (Table 1), which indicated that the boron is not lost during the adsorption and eluting, and all the boron could be recovered completely by the resins. Comparing the values measured by ICP-OES with those by ICP-MS, they were found to be consistent (Figure 1) and the same as the contents of boron in original solutions.

The recoveries of boron in solutions under different salinities ranged from 99.95% to 103.3% (Table 2), which were consistent with the results of pure solution and showed that the salinity had no effect on the adsorption of boron-specific resin and mixed resins. The results demonstrated that the high salt matrix interference of the solution could be excluded effectively, which is suitable for the detection of the boron content by ICP-OES and ICP-MS after removing the high-salt matrix and enriching the boron element. And this method provided a good way to measure the boron content in halite.

The recovery of additional standard of the natural halite (ZK-04, ZK-10) ranged from 98.8% to 106.00% (Table 3), which showed that all the boron of the natural halite was recovered completely by resins. The dissolution of the halite in water, as well as the enrichment and removal of the matrix in the resins, did not result in the loss of boron. There was no difference between the boron contents of the halite measured by ICP-OES and ICP-MS.

### 3.2. Separating Effect by Resins

In order to discuss the separating effect between boron element and foreign ions, three natural samples and four synthetic brines were processed based on the above method. The amounts of foreign ions were measured, whose results (Table 4) showed that the foreign ions in the original solution were removed completely and the relative content of boron reached above 97%. Thus this method was great for the detection of boron content by ICP-OES and ICP-MS.

### 3.3. The Detection Identifications of Boron Content by ICP-OES and ICP-MS

Take the deionized water through the entire process as a blank solution. Perform 11 consecutive measurements, and define 3 times standard deviation of the measurement results as the detection identification, 2 times the detection identification as the identification limit, and 10 times the standard deviation as the quantification limit for boron element [19]. All the parameters were showed in Table 5. If the salinity of the solution was reduced just by dilution, it would cause the boron content of the solution to be lower than the detection limit and quantification limit of ICP-OES and ICP-MS.

### 3.4. The Method Repeatability Test

Repeatedly test solutions at different salinities 6 times, whose results which were showed in Table 6 indicated that the standard deviation ranged from 1.4% to 3.4%, and all the values were less than 5%. So we think that the method is feasible and there is no accidental error for the boron content determination of halite by ICP-OES and ICP-MS.

### 3.5. The Boron Contents of Natural Samples

**Figure 2:**
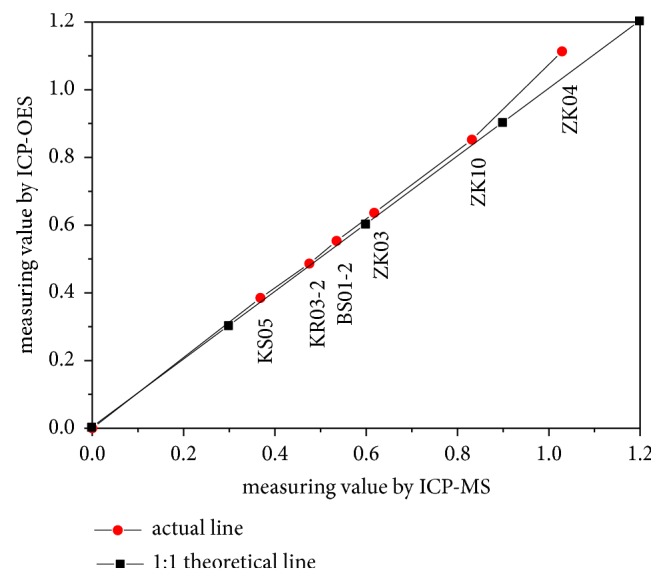
The boron content of natural halite.

The boron contents of natural samples which were showed in Figure 2 ranged from 0.3684 mg·L^−1^ to 1.029 mg·L^−1^, the measuring values of which were beyond the identification limits and the quantification limits of ICP-OES and ICP-MS. The values of natural halite measured by ICP-OES were consistent with those by ICP-MS. Conversely, if only dilution was performed, the boron content in the solution would be similar to the detection limit and the quantitation limit of the instrument; therefore the dilution method could not be applied for the detection of boron content of the halite by ICP-OES and ICP-MS. Comparing the boron content of the halite of Long-hu Diggings, Laos (ZK-03, ZK-04, ZK-10), with that of the salt lake in Pakistan (KR03-2, BS01-2, KS05), we found that the boron contents in Laos halite were higher than those in Pakistan halite. This result was consistent with the geological phenomenon that there are borate mineral inclusions in Long-hu Diggings, Laos [20, 21].

## 4. Conclusion

The boron-specific resin could significantly enrich the boron in high-salt solution and remove the matrix, which is suitable for the detection of the boron content of halite by ICP-OES and ICP-MS, whose standard addition recovery ranged from 97.5% to 106.0, and the relative standard deviations of the repeated experiments were less than 5%.

## Figures and Tables

**Table 1:** The recoveries of boron in pure solutions.

Total amount of boron /*μ*g	Total recovery amount of boron /*μ*g	Recovery /%	
10	9.76	97.6	ICP-OES
30	30.27	100.9	
50	49.60	99.20	
70	71.64	102.34	

10	10.2	102	ICP-MS
30	30.0	100	
50	49.4	98.8	
70	70.3	100.4	

**Table 2:** The recovery results of boron in different salinities.

The content of Na^+^ mg·L^−1^	amount of boron /*μ*g	Measuring by ICP-OES mg·L^−1^	Measuring by ICP-MS mg·L^−1^	Recovery for ICP-OES %	Recovery for ICP-MS %
500	40	4.053	4.132	101.33	103.3
5000	40	4.112	4.023	102.8	100.57
50000	40	4.181	3.998	104.5	99.95

**Table 3:** The recovery additional standard for natural sample.

Sample number	Measuring method	Boron content of original solution mg·L^−1^	The amount of addition standard /*μ*g	Boron content of additional standard solution mg·L^−1^	Recovery additional standard %
ZK-04	ICP-OES	1.029	10	2.089	106.00
	ICP-MS	1.075	10	2.129	105.40

ZK-10	ICP-OES	0.832	10	1.82	98.8
	ICP-MS	0.803	10	1.842	104.2

**Table 4:** The separation effect by boron-special resin and mixed resins.

Sample	The amount of ions /*μ*g				Total amount of foreign ions /*μ*g	The amount of boron /*μ*g	The relative content /%
	Na^+^	Li^+^	Mg^2+^	Cl^−^			

Big Qaidam	0.2	0	0.08	0.13	0.41	24.3 (24.1)	98.3(98.3)
Inter-crystalline Brine	0.26	0.45	0.04	0.32	1.07	48.6(48.3)	97.9(97.8)
Lake water	0.17	0	0.05	0.25	0.47	14.9(15.1)	97.0(96.98)
Synthetic brine	0.08	0	0.02	0.1	0.2	17.6(17.5)	98.9(98.87)
Brine-500	0.1	0	0	0.3	0.4	40.85(40.3)	99.0(99.02)
Brine-5000	0.2	0	0	0.4	0.6	41.25(40.9)	98.57(98.55)
Brine-50000	0.3	0	0	0.7	1.0	41.58(40.7)	97.65(97.6)

*∗*The data in the parentheses represented the results of measuring by ICP-MS, and the other data represented the results of measuring by ICP-OES.

**Table 5:** The detection identification and quantification limits.

Method	Wavelength nm	Average content mg·L^−1^	SD mg·L^−1^	RSD %	detection identification mg·L^−1^	identification limit mg·L^−1^	quantification limit mg·L^−1^
ICP-OES	208.900	-0.002	0.002	3.6	0.006	0.012	0.02

	analytical isotope	Average content mg·L^−1^	SD mg·L^−1^	RSD %	detection identification mg·L^−1^	identification limit mg·L^−1^	quantification limit mg·L^−1^

ICP-MS	^11^B	0.003	0.012	2.8	0.036	0.072	0.12

**Table 6:** Repeated results under different salinities.

number	Na^+^ 500 mg·L^−1^		Na^+^ 5000 mg·L^−1^		Na^+^ 50000 mg·L^−1^	
	By ICP-OES	By ICP-MS	By ICP-OES	By ICP-MS	By ICP-OES	By ICP-MS
1	4.417	4.374	4.231	4.500	4.387	4.407
2	4.165	4.357	4.408	4.396	4.208	4.385
3	4.363	4.246	4.336	4.626	4.547	4.677
4	4.255	4.334	4.249	4.413	4.227	4.743
5	4.425	4.275	4.438	4.369	4.468	4.646
6	4.437	4.385	4.365	4.325	4.556	4.458
average value (mg·L^−1^)	4.344	4.329	4.338	4.438	4.440	4.553
SD	0.11	0.06	0.08	0.11	0.15	0.15
RSD (%)	2.5	1.4	1.8	2.5	3.4	3.3

## Data Availability

The data used to support the findings of this study are included within the article.
